# Derlin1 functions as an oncogene in cervical cancer via AKT/mTOR signaling pathway

**DOI:** 10.1186/s40659-019-0215-x

**Published:** 2019-02-27

**Authors:** Ling Li, Ming Liu, Zhihu Zhang, Wei Zhang, Naifu Liu, Xiugui Sheng, Ping Wei

**Affiliations:** 1 Department of Oncology, People’s Hospital of Tengzhou City, Tengzhou, 277599 Shandong China; 2 Department of Gynecologic Oncology, Shandong Tumor Hospital and Institute, No. 440, Yan Ji Road, Jinan, 250117 Shandong China; 3 Shandong Academy of Occupational Health and Medicine, Jinan, 250062 Shandong China; 4 Department of Obstetrics and Gynecology, Affiliated Hospital of Binzhou Medical College, Binzhou, Shandong China; 5 Department of Gynecologic Oncology, Tumor Hospital of Chinese Academy of Medical Sciences, Shenzhen, 518116 China

**Keywords:** Derlin1, Cervical cancer, Bcl-2, Bax, AKT/mTOR

## Abstract

**Background:**

Cervical cancer (CC) ranks third in the morbidity and mortality of female cancer around the world. Derlin1 has been found to be overexpressed in several human cancers. However, it is still unclear about its roles in CC. The research aims to explore the relationship between Derlin1 and CC.

**Methods:**

We purchased a human CC tissues microarray, which contained CC tissues and corresponding para-cancerous tissues from 93 patients with primary cervical squamous cell carcinoma. Immunohistochemical staining was used to confirm the expression of Derlin1 in these tissues. And we detected the differential expression of Derlin1 in cervical cancer cell lines and normal cervical epithelial cells (H8). Further, the cervical cancer cell lines SiHa and C33A were used as an in vitro model, which was down-regulated the expression of Derlin1 using siRNA interference technology. The effects of Derlin1 down-regulating in CC cell lines on cell proliferation and migration were detected by CCK8 assay and transwell assay, respectively. The effect of Derlin1 down-regulating on apoptosis was analyzed by flow cytometry, and apoptosis-related proteins were detected using western blotting. In-depth mechanisms were studied using western blotting. In addition, the effects of Derlin1 up-regulating in normal cervical epithelial cells also were exposed.

**Results:**

Derlin1 was significantly elevated in CC tissues (81.7%, 76/93), and the expression of Derlin1 was positively correlated with the tumor size, pathological grade, and lymph node metastasis in CC patients. And Derlin1 was high expressed in cervical cancer cell lines compared to H8 cells. Knockdown of Derlin1 in cervical cancer cell lines inhibited cell proliferation and migration. Moreover, knockdown of Derlin1 induced apoptosis and affected the expression of apoptosis-related proteins, including Bcl-2, Bax, Bim, caspase3 and caspase9. Further experiments showed that AKT/mTOR signal pathway might be involve in this processes that knockdown of Derlin1 inhibited the expression of p-AKT and p-mTOR. Over-expression of Derlin1 in H8 cells promoted cell proliferation and migration via up-regulated the expression of p-AKT and p-mTOR.

**Conclusion:**

Derlin1 is an oncogene in CC via AKT/mTOR pathway. It might be a potential therapeutic target for CC.

## Introduction

Cervical cancer (CC) ranks third in the morbidity and mortality of female cancer around the world [1]. The annual incidence of CC worldwide is over 450,000, of which more than 80% occur in developing countries [2]. CC is a malignant tumor that mostly occurs at the junction of the cervical scale column, and is highly invasive and metastatic. The standard treatment for CC includes early surgery, chemotherapy, and advanced radiation therapy [3]. However, clinical outcomes between patients are significantly different, and difficult to predict [4]. Although the morbidity and mortality of CC have declined over the past decade, and significant advances has been made in prevention, but there has been few new breakthroughs in prognosis prediction [4]. Therefore, it is very important to improve treatment strategies and expand the molecular indicators of prognosis.

Derlin1 is a 22 kDa endoplasmic reticulum (ER) membrane protein with four or six transmembrane, and responsible for transporting unfolded or misfolded proteins from the ER lumen to the cytoplasm [5]. Subsequently, these proteins are degraded by the ubiquitin proteasome or autolysosome in cytoplasm [6]. A recent study find that Derlin1 inhibits the protein expression of epithelial Na^+^ channel (ENaC), and promotes its ubiquitin degradation [7]. In recent years, there is increasing evidences that the expression of Derlin1 is closely related to the tumorigenesis and progression. Derlin1 is highly expressed in several types of cancer, including breast cancer, lung cancer, colon cancer, bladder cancer, esophageal squamous cell carcinoma (ESCC), and head and neck squamous cell carcinoma (SCCHN) [8–11]. The high expression of Derlin1 in breast and lung cancers is associated with tumor grade and lymph node metastasis [6, 10]. The expression of Derlin1 in muscle invasive bladder cancer is higher than that in non-muscle invasive bladder cancer [11]. Derlin1 antibodies inhibit tumor growth in a mouse model of colon cancer [6]. Derlin1 knockdown in bladder cancer also has been confirmed to inhibit cell migration [8]. However, little is known about the action mechanism of Derlin1 in CC.

This study investigated the expression of Derlin1 in CC and further explored the effect of Derlin1 knockdown on cervical cancer cell lines SiHa and C33A. We found that knockdown of Derlin1 inhibited the cell proliferation and migration, promoted apoptosis. AKT/mTOR signaling pathway may participate in this process. Derlin1 may be a target for therapy and prediction in CC.

## Materials and methods

### Tissue samples

Human CC tissues microarray was purchased from ShGnghGi Outdo Biotech CompGny (OD-CT-RpUtr-03, Shanghai, China), including CC tissues and corresponding para-cancerous tissues from 93 patients with primary cervical squamous cell carcinoma. The para-cancerous was cervical epithelia within 3 cm of the focus. The clinicopathological data of CC patients included age, tumor size, tumor site, lymph node metastasis, and clinical stages, summarized in Table 1.

**Table 1:** Derlin1 expression associated with the clinicopathological parameters in CC

Clinicopathological parameters	n	Derlin1 high (n%)	Derlin1 low (n%)	p
Age (years)				
< 50	71	56 (78.9)	15 (21.1)	0.201876
≥ 50	22	20 (90.9)	2 (9.1)	
Tumor diameter (cm)				
< 3	43	29 (67.4)	14 (32.6)	0.000953*
≥ 3	50	47 (94.0)	3 (6.0)	
Lymph node metastasis				
Yes	59	52 (88.1)	7 (11.9)	0.034982*
No	34	24 (70.6)	10 (29.4)	
Pathological grading				
I–II	31	21 (12.9)	10 (87.1)	0.037635*
III–IV	62	55 (56.5)	9 (43.5)	

* P < 0.05

### Immunohistochemistry

Cervical cancer tissues microarrays were stained using the EliVisionTM plus kit (Maixin, China) according to the manufacturer’s instructions [12]. Anti-Derlin1 (rabbit polyclonal antibody) was purchased from GeneTex (GTX48541, USA). The Derlin1 immunostaining score was the sum of the staining intensity score and the positive staining cell rate score. The staining intensity was scored as follows: no staining (0); weak staining (1); moderate staining (2); and strong staining (3). The positive staining cell rate was scored as follows: 0 to 5% (0); 5% to 25% (1); 26% to 50% (2); 51% to 75% (3) and > 75% (4). A score below 2 points was considered to be Derlin1 low expression, > 3 points as Derlin1 high expression.

### Cell culture and transfection

The cervical cancer cell lines (SiHa, C33A, Caski and C4-1) and cervical epithelial immortalized cells (H8) were purchased from the Cell Resource Database of Chinese Academy of Sciences (Shanghai, China). Cells were routinely cultured in high glucose DMEM medium supplemented (Thermo Fisher Scientific, Waltham, MA, USA) with 10% fetal bovine serum (FBS) (Gibco, Carlsbad, CA, USA), 100 U/ml penicillin (Sigma-Aldrich, St. Louis, MO, USA), and 0.1 mg/ml streptomycin (Sigma-Aldrich, St. Louis, MO, USA) at 37 °C in 5% CO_2_. When the cell density reached approximately 60%, the cells were transfected with Derlin1 siRNA or Derlin1-overexpressed plastic using Lipofectamine 2000. A nonsense siRNA or plastic were transfected into cells as a negative control (NC), and normally cultured cells as control (CON). Derlin1 siRNA and overexpressed plastic were obtained from Ruibo (Guangzhou, China).

### Real-time quantitative PCR

Total RAN was extracted from cells after 24 h of transfection using Trizol reagent (Invitrogen, Carlsbad, CA, USA), and then reverse transcribed to cDNA by the RevertAid First Strand cDNA Synthesis Kit (TakaraBio, Otsu, Japan). The Derlin1 mRNA expression was performed by SYBR Premix Ex Taq II (TaKaRa, Dalian, China). The relative quantification of Derlin1 was determined using the 2^−∆∆Ct^ method after normalization to the GAPDH. Primers used were as follows: Derlin1 sense: 5′-CGCTTTCAGATTTGGAGGCC-3′, anti-sense: 5′-GCCTCCCATCAAAAGCTCCT-3′; GAPDH sense: 5′-GACTTCAACAGCGACACCCA-3′, antisense: 5′-CACCCTGTTGCTGTAGCCAAA-3′.

### Western blot and antibody

For cells transfected for 48 h, cells were lysed with ice-cold RIPA buffer (ComWin Biotech, Beijing, China) containing protease inhibitors and phosphatase inhibitors (ComWin Biotech, Beijing, China). Cell lysates were collected from culture plates, and total proteins were collected by centrifugation. Protein concentrations were quantified using Bio-Rad protein assay kit (ComWin Biotech, Beijing, China). Proteins were boiled in 4× protein loading buffer for 5 min, followed by an ice bath for 5 min. The proteins (15 μg) were separated by SDS-PAGE and transferred to a PVDF membrane. Then, the membrane was preblocked, and incubated overnight at 4 °C with primary antibodies, followed by the secondary antibodies (1:2000) at room temperature for 1 h. The chemiluminescent signals of proteins were generated by ECL reagent, and quantified with QUANTITY ONE software.

The primary antibodies used in this study were as follow: anti-Derlin1 (1:1000, Rabbit polyclonal antibody, GTX48541, GeneTex, USA); anti-Bcl-2 (1:2000, Mouse polyclonal antibody, 60178-1-Ig, Proteintech, Manchester, UK); anti-Bax (1:1000, Rabbit polyclonal antibody, 50599-2-Ig, Proteintech, Manchester, UK); anti-Bim (1:3000, Rabbit polyclonal antibody, DF6093, Affinity Biosciences, OH, USA); anti-caspase3 (1:1000, Rabbit polyclonal antibody, 19677-1-AP, Proteintech, Manchester, UK); anti-caspase9 (1:1000, Rabbit polyclonal antibody, 10380-1-AP, Proteintech, Manchester, UK); anti-p-AKT (1:1000, Mouse monoclonal antibody, Ser473, 66444-1-Ig, Proteintech, Manchester, UK); anti-AKT (1:500, Mouse monoclonal antibody, #9272, Cell Signaling Technology, Beverly, MA); anti-p-mTOR (1:1000, Mouse monoclonal antibody, Ser248, #5536, Cell Signaling Technology, Beverly, MA); anti-mTOR (1:1000, Mouse monoclonal antibody, #2972, Cell Signaling Technology, Beverly, MA).

### CCK8 assay

For CCK8 assay, 5 × 10^3^ cells that transfected for 24 h, were seeded onto 96-well plate. After cultured for 24 h, 48 h, 72 h, 100 μl spent medium was replaced with an equal volume of fresh medium containing 10 μl CCK8, and cells was incubated for 2 h at 37 °C. The proliferation of cells was determined by measuring OD value at 450 nm using microplate spectrophotometer.

### Transwell migration assay

Cells (1 × 10^5^) that transfected for 24 h, were seeded in the upper chamber of boyden chamber (8 μm pore size) (Millipore, Billerica, MA, USA) with 100 μl high glucose DMEM medium. Then 600 μl medium with 10% FBS was added to the lower chamber to guide cell migration. After incubation for 24 h, the residual cells on the upper chamber were removed. The migrated cells were fixed by 4% paraformaldehyde for 30 min, stained with 0.1% crystal violet for 20 min, and photographed under the microscope.

### Apoptosis detection

Cell apoptosis was analyzed using Annexin V-FITC Apoptosis Detection kit I (4A Biotech, Nanjing, China). After transfected for 48 h, cells were harvested and washed with precooled PBS. Annexin V binding buffer was added to resuspend cells to 1–5 × 10^6^/ml. 100 μl cell suspension was incubated with 5 μl Annexin V/FITC mix for 5 min. Then 10 μl PI dye and 400 μl PBS were added, and apoptosis was detected by flow cytometry. Statistical analysis was performed using Flowjo software (Tree Star, Ashland, OR, USA).

### Statistical analysis

SPSS 20.0 statistical analysis software was used to analyze the experimental data. The Pearson Chi Square analysis was used to analyze the correlation between expression of Derlin1 and clinicopathological characteristics of CC patients. Measured data were expressed as mean ± standard deviation (SD). Student’s t-test was used for comparison between the two groups, and one-way ANOVA was performed to compare three or more groups, and *p* < 0.05 was considered statistically significant.

### Patient and public involvement

Patient or public involvement in this research was not required as the data from ShGnghGi Outdo Biotech CompGny (OD-CT-RpUtr-03, Shanghai, China).

## Results

### Expression of Derlin1 and its correlation with clinical parameters in CC patients

**Fig. 1:**
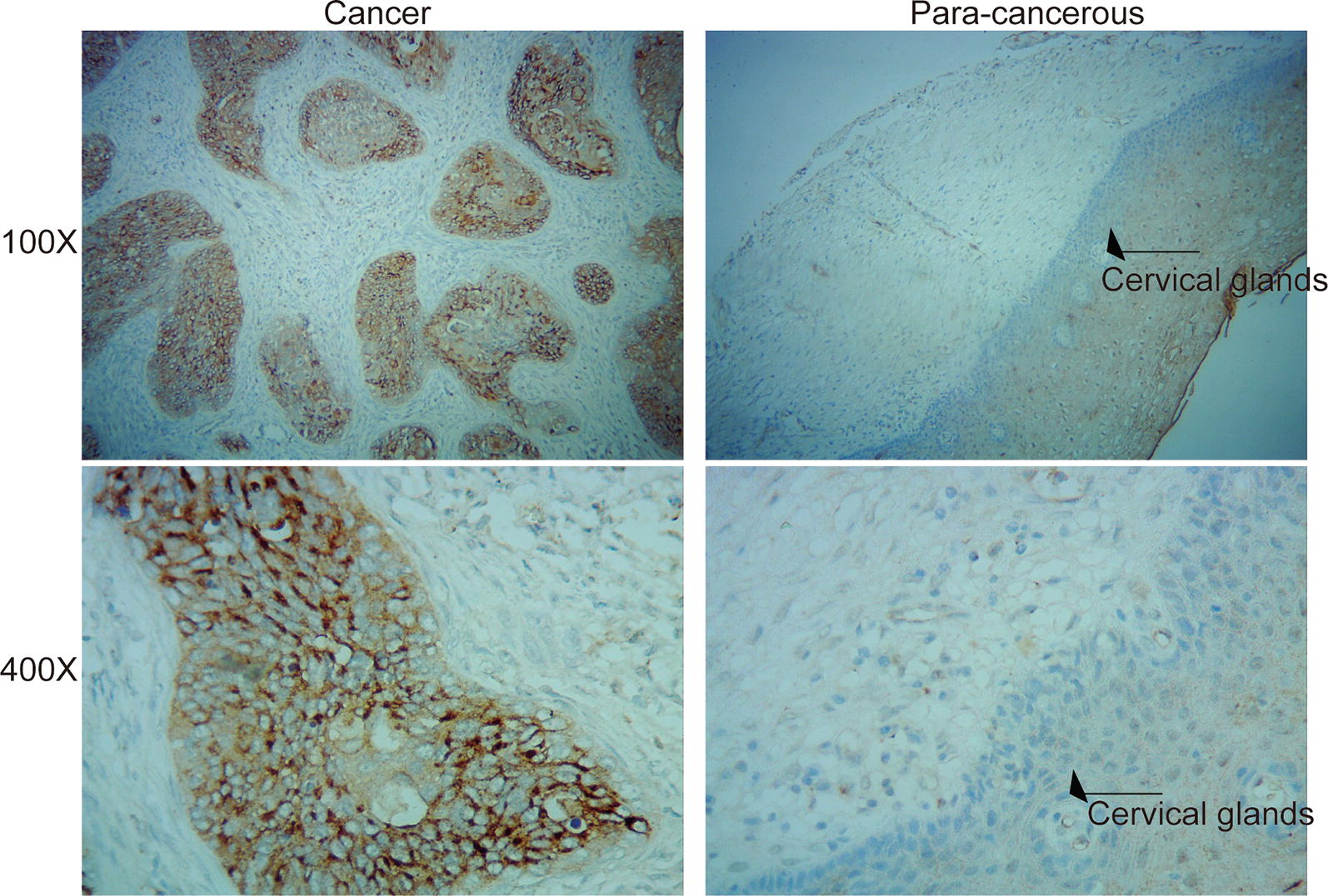
Derlin1 is up-regulated in CC. Immunohistochemical staining of Derlin1 in HCC tissues and para-carcinoma

To investigate the clinical significance of Derlin1 in CC, we first detected the expression of Derlin1 by immunohistochemistry in 96 pairs of cervical tumors and corresponding paracancerous tissue. As shown in Fig. 1, Derlin1 positive signal was mainly located in the CC cytoplasm. And Derlin1 was highly expressed in 81.7% of CC tissues (76/93), and was highly expressed only in 12.9% of corresponding paracancerous tissue (12/93, *p *< 0.001, Table 2). This result suggest that expression of Derlin1 is up-regulated in CC compared to paracancerous tissue. Table 1 showed the correlation analysis between Derlin1 expression and CC clinical characteristics. As expected, the high Derlin1 expression showed higher incidences of larger tumors sizes (*p* = 0.000953), lymph node metastasis (*p * = 0.034982), high pathological grade (*p * = 0.037635). In addition, the expression of Derlin1 in CC was not related to age (*p * = 0.201876). Taken together, these finding indicate that Derlin1 might correlate the tumor growth and metastasis of CC.

**Table 2:** Derlin1 expression in CC and para-carcinoma tissue

Group	n	Derlin1 expression		p
		Low (n%)	High (n%)	
CC	93	17 (18.3)	76 (81.7)	< 0.001*
Para-carcinoma	93	81 (87.1)	12 (12.9)	

### Knockdown of Derlin1 inhibits the proliferation and migration of CC cells

**Fig. 2:**
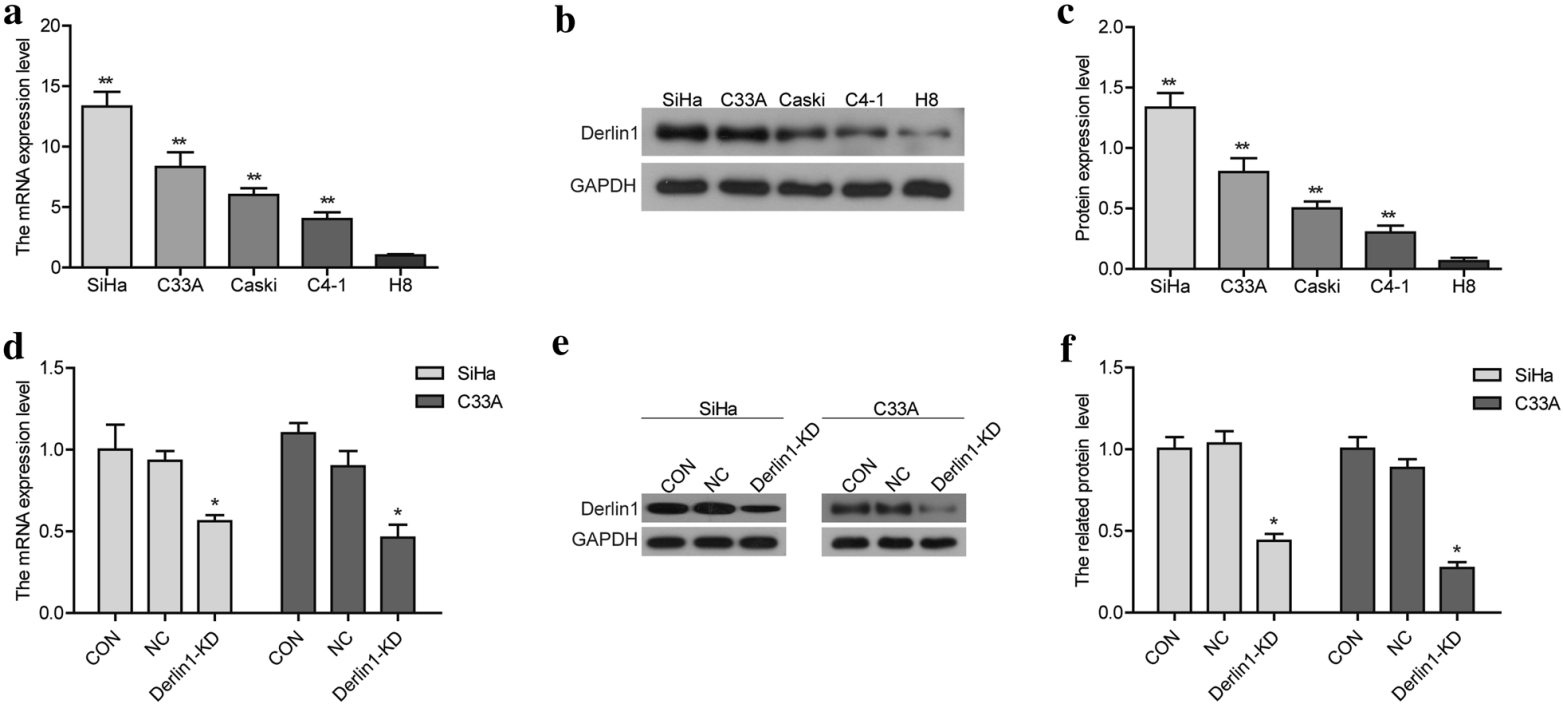
The expression of Derlin1 is knocked down using Derlin1-siRNA. **a**–**d** The mRNA levels of Derlin1 were detected by qRT-PCR. **b**–**e** The protein levels of Derlin1 were detected by western blot. **c**–**f** Quantitative results of protein expression levels. N = 3, **p < 0.01 compared to H8, *p < 0.05 compared to NC

Firstly, we detected the expression of Derlin1 in cervical cancer cell lines (SiHa, and C33A, Caski and C4-1) and cervical epithelial immortalized cells (H8) by using qRT-PCR and western blot. As shown in Fig. 2a–c, Derlin1 was high expressed in cervical cancer cell lines, compared to H8 cells. And Derlin1 was highest expressed in SiHa and C33A cells. Thus, human cervical cancer cell lines SiHa and C33A were used as a model in vitro, and siRNA were designed to knockdown the expression of Derlin1 in cells (Derlin1-KD). Figure 2d–f showed the interference efficiency of Derlin1. From the results, the transfection of Derlin1-siRNA significantly down-regulated the mRNA and protein expression of Derlin1 (*p *< 0.05), compared to CON and NC.

**Fig. 3:**
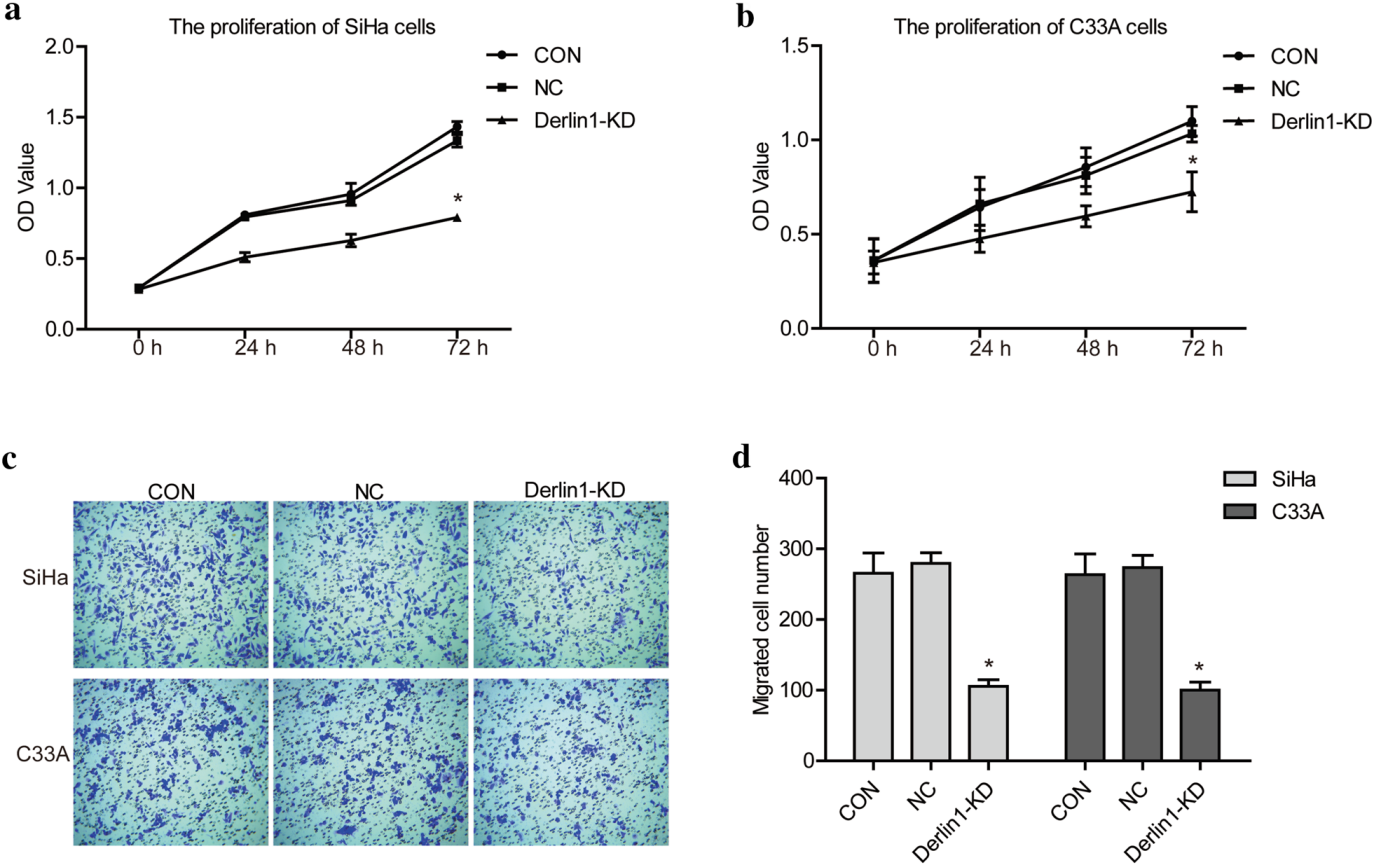
Knockdown of Derlin1 inhibits the proliferation and migration of CC cells. **a**, **b** CCK8 assay showed that Derlin1 knockdown inhibited cell proliferation activity. **c** Transwell assay showed that Derlin1 knockdown inhibited cell migration activity. **d** Quantitative results of migration cell number. N = 3, *p < 0.05 compared to NC

CCK8 assay was performed to confirm the difference of proliferation capacity between CON, NC and Derlin1-KD cells. As shown in Fig. 3a, b, the OD value of Derlin1-KD cells was decreased in comparison to CON and NC cells, and the difference was statistically significant after transfection 72 h (*p *< 0.05). The results demonstrate that the knockdown of Derlin1 inhibits the proliferation of SiHa and C33A cells.

Follow, the difference in migration ability was detected using transwell assay between CON, NC and Derlin1-KD cells. The results were shown in Fig. 3c, d, the migration cell number of Derlin1-KD cells was significant reduced, compared to CON and NC cells (*p *< 0.05). Our results indicate that the knockdown of Derlin1 impede the migration of CC cells. This discovery was encouraging. Because after receiving standard treatment, the 5-year survival rate of cervical cancer patients with no metastatic is 80–90%; while the 5-year survival rate of patients with metastatic cervical cancer is 30–35%. Derlin1 provides a new target for the protection of cervical cancer metastasis.

In summary, the knockdown of Derlin1 inhibits the proliferation and migration of SiHa and C33A cells, which suggest that Derlin1 may play an oncogenic role in CC.

### Knockdown of Derlin1 induces the apoptosis of CC cells

**Fig. 4:**
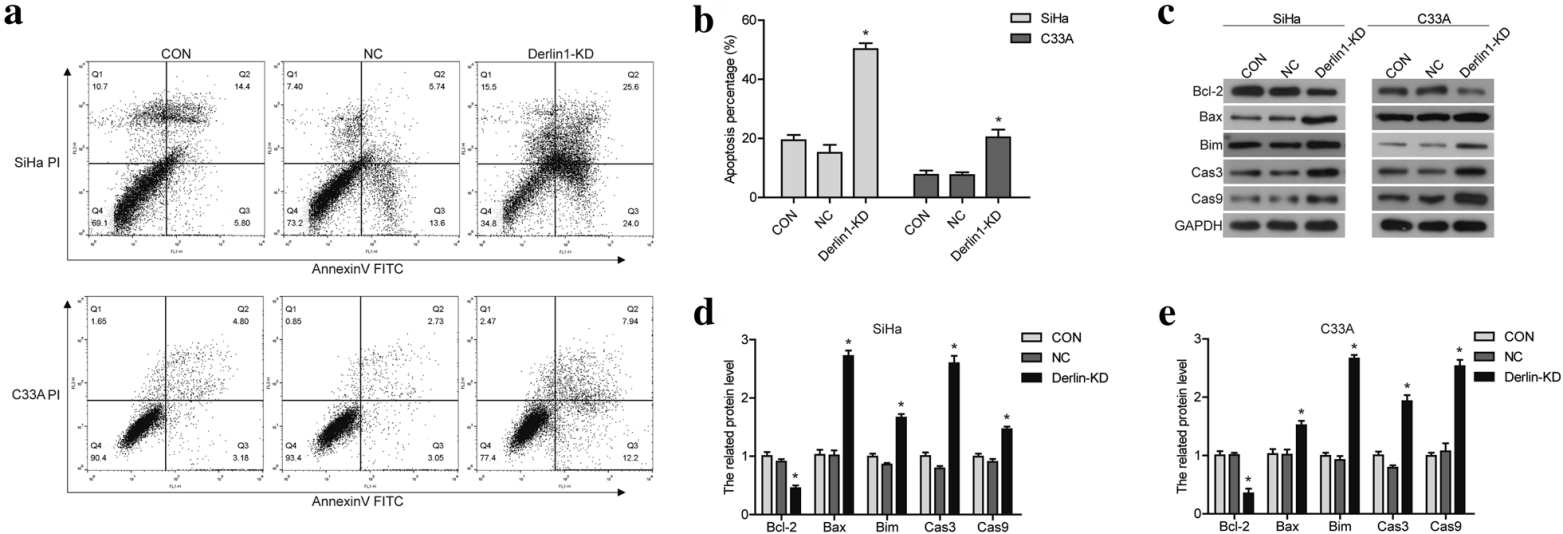
Knockdown of Derlin1 induces the apoptosis of CC cells. **a** Apoptosis was detected by flow cytometry. **b** Quantitative results of apoptotic cell percentage. **c** The proteins expression of apoptosis-related proteins were detected by western blot. **d** Quantitative results of protein expression levels. N = 3, *p < 0.05 compared to NC. cas3: caspase3; cas9: caspase9

We investigated the role of Derlin1 in SiHa and C33A cells apoptosis by flow cytometry analysis. As shown in Fig. 4a, b, apoptosis rate of Derlin1-KD cells increased significantly compared with CON and NC cells (*p *< 0.05). We also detected the expression of apoptotic associated proteins. As shown in Fig. 2c–e, the expression of pro-apoptosis proteins, Bax, Bim, caspase3 and caspase9, were up-regulated after Derlin1-siRNA transfection; and the expression of anti-apoptosis protein Bcl-2 was down-regulated. Taken together, the knockdown of Derlin1 induced the apoptosis of SiHa and C33A cells.

### Knockdown of Derlin1 inhibites AKT/mTOR pathway in CC cells

**Fig. 5:**
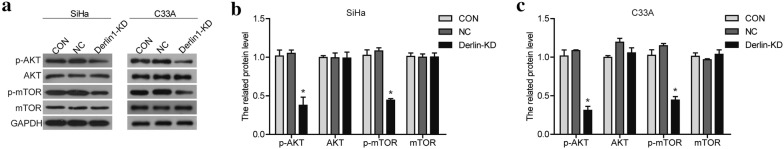
Knockdown of Derlin1 inhibited AKT/mTOR pathway in CC cells. **a** The proteins expression of AKT, p-AKT, mTOR and p-mTOR were detected by western blot. **b**, **c** Quantitative results of protein expression levels. N = 3, *p < 0.05 compared to NC

The signaling pathway regulated by Derlin1 was analyzed in SiHa and C33A cells. The results were shown in Fig. 5, the p-AKT and p-mTOR levels were significantly decreased in Derlin1-KD cells compared to CON and NC cells; while the expression of AKT and mTOR weren’t impacted. These data suggest that Derlin1 knockdown influence the phosphorylation of AKT and mTOR in SiHa and C33A cells.

### Over-expression of Derlin1 induces the malignant phenotypes of H8 cells

**Fig. 6:**
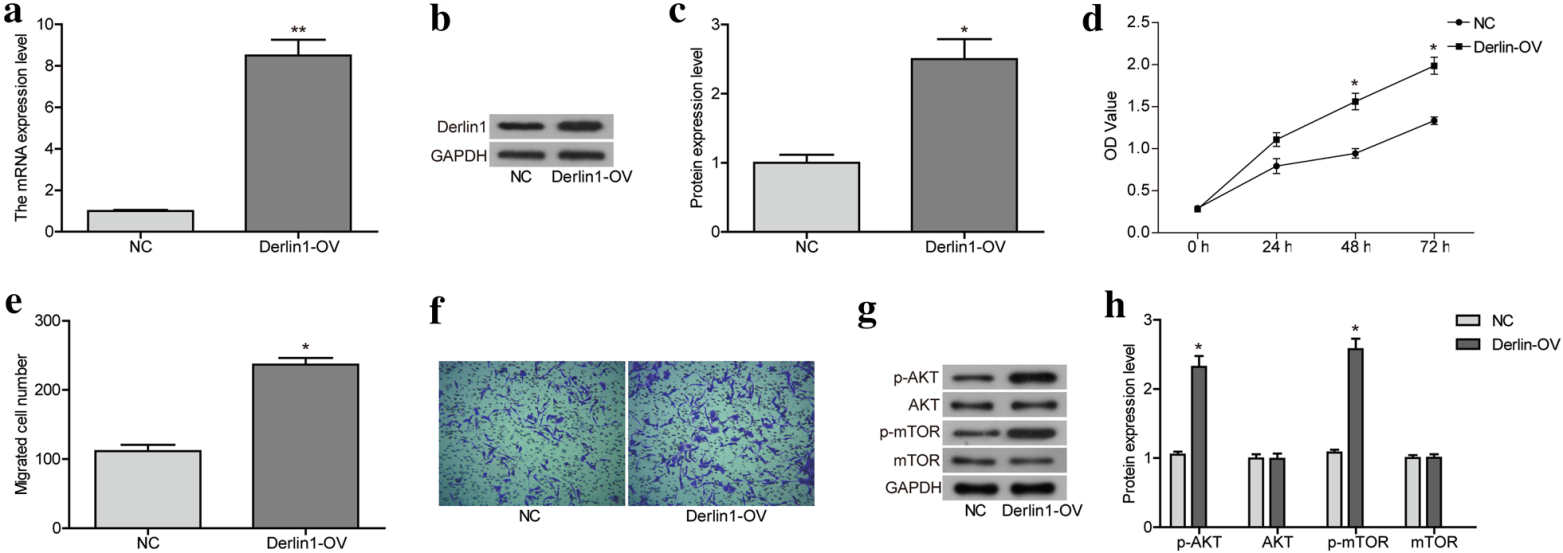
Over-expression of Derlin1 induces the malignant phenotypes of H8 cells. **a** The mRNA levels of Derlin1 were detected by qRT-PCR. **b** The protein levels of Derlin1 were detected by western blot. **c** Quantitative results of protein expression levels. **d** CCK8 assay showed that over-expression of Derlin1 promoted the cell proliferation activity. **e** Quantitative results of migration cell number. **f** Transwell assay showed that over-expression of Derlin1 promoted cell migration activity. **g** The proteins expression of AKT, p-AKT, mTOR and p-mTOR were detected by western blot. **h** Quantitative results of protein expression levels. N = 3, **p < 0.01 compared to NC, *p < 0.05 compared to NC

Our previous research showed that Derlin1 played an oncogene role in CC cells, and knocking down its expression can inhibit the malignant phenotype of CC cells. Therefore, we next wanted to verify whether over-expression of Derlin1 in normal cervical epithelial cells could induce malignant phenotypes. As shown in Fig. 6a–c, the transfection of Derlin1-overexpressed plastic significantly up-regulated the mRNA and protein expression of Derlin1 (*p *< 0.05, Derlin1-OV) compared to NC. And the over-expression of Derlin1 promoted the proliferation and migration of H8 cells (Fig. 6d–f). Further, the over-expression of Derlin1 up-regulated the expression of p-AKT and p-mTOR, not influenced the AKT and mTOR levels (Fig. 6g, h). These results indicate that over-expression of Derlin1 is associated with the malignant phenotype of normal cervical epithelial cells.

## Discussion

As the tumor grows, tumor cells secrete angiogenic factors that promote angiogenesis to ensure the oxygen and nutrients supply of tumor [13]. However, the lack of oxygen and nutrients remains a major feature of the tumor microenvironment [14]. Hypoxia and glucose deprivation may induce ER stress [14]. Upon ER stress, unfolded or misfolded proteins in the ER lumen are translocated to the cytoplasm by the transporter, then degraded by the ubiquitin proteasome or autolysosome system [15]. If ER stress has not been effectively alleviated, cells will initiate multiple apoptotic pathways that eventually lead to cell death [16]. Almost all solid tumors have ER stress; it is still unclear that how tumors respond to long-term ER stress [8].

Derlin1 may be involved in the tumor’s response to ER stress [5]. In ER stress, Derlin1 expression is enhanced, which promotes the translocation of unfolded or misfolded proteins and inhibits apoptosis [6]. Evidences confirm that Derlin1 is involved in the progression of multiple tumors. Derlin1 attenuates ER stress- induced apoptosis in breast cancer cells [10]. Derlin1 is highly expressed in bladder cancer tissues and bladder cancer cell lines [8], and its overexpression induces cisplatin tolerance in bladder cancer patients [11]. The survival rate of bladder cancer patients with Derlin1 positive expression is significantly shorter than that of negative patients [11]. Up-regulation of Derlin1 reverses the tumor suppressive effect of miR-181d on ESCC [11]. However, the expression and role of Derlin1 in CC remains unclear. In this study, we performed an immunohistochemical assay to assess the expression of Derlin1 in CC and paracancerous tissues. We found that Derlin1 expression was significantly up-regulated in CC. In addition, the expression of Derlin1 was positively correlated with tumor size, pathological grade, and lymph node metastasis in CC patients. These results indicate that Derlin1 may act as an oncogene in CC progression. In vitro studies further support this argument. We found that Derlin1 was high expressed in cervical cancer cell lines compared to H8 cells. And knockdown of Derlin1 in SiHa and C33A cells inhibited cell proliferation and migration. It shows that Derlin1 may be a new therapeutic target for CC.

In this study, we found that inhibiting the Derlin1 expression in SiHa and C33A cells strongly induced apoptosis and affected the expression of apoptosis-related proteins, including Bcl-2, Bax, Bim, caspase3 and caspase9. Bcl-2 and Bax are the members of Bcl-2 superfamily, and are involved in apoptosis by modulating mitochondrial membrane potential (MMP) and the release of cytochrome C [16]. Bax is an important pro-apoptosis protein [17]. And Bcl-2 can insert into various intracellular membranes to antagonize Bax protein [18]. Several studies have shown that the levels of Bcl-2 and Bax in CC has a close correlation with tumor stage, radiosensitivity, and prognosis [19]. Through the immunohistochemistry of Bcl-2 in cervical intraepithelial neoplasia and invasive squamous cell carcinoma, Saegusa et al. find that Bcl-2 is associated with the early occurrence of CC [19]. Mukherjee et al. compare the immunohistochemistry staining of Bcl-2 in recurrent and primary cervical cancer tissues, and find that the Bcl-2 positive expression is related to the 5-year survival rate [20]. It has been reported that the overexpression of Bcl-2 in various cervical cancer cell lines enhances the resistance to radiation [21]. Bcl-2 is closely related to radiotherapy sensitivity and prognosis of CC patients after radiotherapy. Overexpression of Bax in various cervical cancer cell lines and primary cervical cancer cells induces robust apoptosis [22]. At present, the expression of Bcl-2 and Bax has been used as a prognostic indicator for CC patients after radiotherapy [23]. Our results indicate that Derlin1 could be used to modulate prognosis of CC by regulating Bcl-2 and Bax.

Further experiments showed that the levels of p-AKT and p-mTOR were decreased when the expression of Derlin1 was reduced in SiHa and C33A cells. A similar research was reported in SCCHN cell lines [11]. Decreased expression of Derlin1 in SCCHN cell lines inhibits the cell proliferation by restrained the phosphorylation of PI3K and AKT [11].

At the last, we found that over-expression of Derlin1 in normal cervical epithelial cells could induce malignant phenotypes, including promoting proliferation and migration via up-regulating the expression of p-AKT and p-mTOR.

## Conclusion

This research demonstrates that Derlin1 is highly expressed in CC. In addition, knockdown of Derlin1 in SiHa and C33A cells could inhibit the cell proliferation and migration, and promote apoptosis. Further, AKT/mTOR signaling pathway may participate in this process. And over-expression of Derlin1 in normal cervical epithelial cells could induce malignant phenotypes via up-regulating the expression of p-AKT and p-mTOR. Taking together, Derlin1 may be a target for therapy and prediction in CC.

## Abbreviations

CC: cervical cancer
ER: endoplasmic reticulum
EnaC: epithelial Na+ channel
ESCC: esophageal squamous cell carcinoma
SCCHN: head and neck squamous cell carcinoma
NC: negative control
CON: control
SD: standard deviation
